# Celiac Disease: A Transitional Point of View

**DOI:** 10.3390/nu17020234

**Published:** 2025-01-10

**Authors:** Ernesto Aitella, Domenico Cozzolino, Lia Ginaldi, Ciro Romano

**Affiliations:** 1 Department of Clinical Medicine, Public Health, Life and Environmental Sciences, University of L’Aquila, 67100 L’Aquila, Italy; ernestoaitella@gmail.com (E.A.); lia.ginaldi@univaq.it (L.G.); 2 Allergy and Clinical Immunology Unit, “G. Mazzini” Hospital, ASL Teramo, 64100 Teramo, Italy; 3 Division of Internal Medicine, Department of Precision Medicine, “Luigi Vanvitelli” University of Campania, 80131 Naples, Italy; domenico.cozzolino@unicampania.it; 4 Clinical Immunology Outpatient Clinic, Division of Internal Medicine, Department of Advanced Medical and Surgical Sciences, “Luigi Vanvitelli” University of Campania, 80131 Naples, Italy

**Keywords:** celiac disease, gluten, immunopathogenesis, dietary management

## Abstract

Celiac disease (CeD) is a chronic, lifelong, multifactorial, polygenic, and autoimmune disorder, characteristically triggered by exposure to the exogenous factor “gluten” in genetically predisposed individuals, with resulting duodenal inflammation and enteropathy, as well as heterogeneous multisystemic and extraintestinal manifestations. The immunopathogenesis of CeD is complex, favored by a peculiar human leukocyte antigen (HLA) genetic predisposition, leading to gluten presentation by antigen-presenting cells to CD4+ T helper (Th) cells, T cell–B cell interactions, and production of specific antibodies, resulting in the immune-mediated killing of enterocytes and, macroscopically, in duodenal inflammation. Here, the most relevant correlations between cellular and molecular aspects and clinical manifestations of this complex disease are reviewed, with final considerations on nutritional aspects for disease management.

## 1. Introduction

Celiac disease (CeD) is a chronic, lifelong, multifactorial polygenic and autoimmune disorder, characteristically triggered by exposure to the exogenous factor “gluten” in genetically predisposed individuals, with resulting duodenal inflammation and enteropathy, as well as heterogeneous multisystemic extraintestinal manifestations [1]. The global prevalence of CeD is estimated to be 0.7% (biopsy-confirmed cases) or 1.4% (considering only positive serology) and is significantly greater in children than adults (0.9% vs. 0.5%) [1,2]. Although abdominal symptoms reflect the characteristic intestinal involvement, a great deal of symptoms are the consequence of extraintestinal manifestations, which may cause significant delays in diagnosis.

Briefly, with regard to the immunopathogenesis of CeD, a peculiar human leukocyte antigen (HLA) constellation favors gluten presentation by antigen-presenting cells (APCs) to CD4+ T helper (Th) cells, T cell–B cell interactions, and production of specific antibodies, resulting in immune-mediated killing of enterocytes and, macroscopically, in duodenal inflammation [3,4].

The purpose of this review is to provide updated knowledge on the relationship between pathogenetic findings and clinical manifestations, with a final focus on the implications impacting the dietary management of this disease.

## 2. From Transglutaminase Isoforms to Clinical Features: Dermatological and Neurological Involvement

Unlike anti-native gliadin (AGA) and anti-deamidated gliadin (DGP) antibodies, a critical role in CeD is played by autoantibodies against tissue transglutaminase (anti-tTG), particularly of the IgA isotype. Detection of anti-tTG IgA, therefore, serves as the main serologic diagnostic tool in both adulthood and childhood forms of CeD, with a strong sensitivity (98%) and specificity (90%), overall greater than 90%, provided selective IgA deficiency is ruled out [5,6,7]. Tissue transglutaminase or transglutaminase 2 (TG2) is part of the protein–glutamine γ-glutamyltransferase family and is one of at least nine such enzymes in humans; it is a calcium-dependent ubiquitously expressed enzyme, which binds to and stabilizes proteins of the extracellular matrix (ECM), particularly fibronectin. TG2 is also involved in cell signalling, cellular apoptosis, or proliferation and motility and catalyzes, among other reactions, the pivotal transamidation and deamidation of gluten proteins enriched in glutamine and proline [8,9,10].

Conversely, the epidermal transglutaminase 3 (TG3) [11] is considered the autoantigen of dermatitis herpetiformis (DH), a characteristic cutaneous lesion observed in up to 10% of CeD patients. DH is caused by IgA against the dermal deposits of TG3, resulting in an itchy blistering symmetrical rash, prevalently on elbows, knees, and buttocks; vesicles appear in clusters and then evolve in eroded and crusted lesions because of scratching [12,13]. The finding of granular IgA deposits in the papillary dermis by direct immunofluorescence or biopsy of perilesional skin is diagnostic. DH is more common in male, middle-aged adults; although it is not usually associated with gastrointestinal symptoms, DH shares with CeD the finding of anti-TG2 IgA autoantibodies in serum and typical histological alterations in the duodenum. DH shows a good but slower response to a gluten-free diet with respect to CeD, necessitating prescription of dapsone in most patients to control skin lesions at onset [14,15].

**Figure 1:**
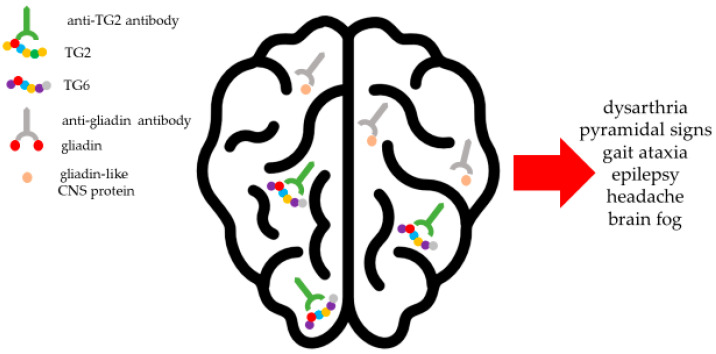
Immunopathogenesis of neurological manifestations. Following breakdown of the brain–blood barrier induced by pro-inflammatory cytokines, circulating anti-transglutaminase (TG)2 and anti-gliadin antibodies may gain access to the central nervous system (CNS), where they may react with structurally similar proteins, i.e., TG6 and CNS proteins sharing sequence homology with gliadin, respectively, leading to brain inflammation and neurological symptoms.

Similarly, the high homology between intestinal TG2 and the brain isoform TG6, as well as molecular mimicry between gliadin and proteins in the central nervous system, underlies the neurological manifestations of CeD (Figure 1). Clinically, neurological involvement may be recorded in up to 36% of adults, even before CeD appearance. The breakdown of blood–brain barrier (BBB) integrity, high concentrations of such chemokines as CXCL10, anti-Purkinje cell antibodies, and expression of TG6 from activated astrocytes, neuroglia, and neuronal cells of critical locomotor brain areas may lead to dysarthria, dysphonia, pyramidal signs, abnormal eye movements, and progressive gait ataxia, the latter also known as “gluten ataxia” [16]. The production of anti-neuronal antibodies and the BBB breakdown driven by pro-inflammatory cytokines, such as interleukin (IL)-1, IL-6, IL-8, and tumor necrosis factor (TNF)-α produced in the small bowel, contribute to the development of peripheral neuropathy, headache, cognitive impairment, “brain fog”, epilepsy, psychiatric symptoms, or states of neurological low chronic inflammation [17].

## 3. Gut–Brain Axis in CeD: The Role of Microbiota

Interestingly, CeD and multiple sclerosis may share a similar pathophysiological model for central nervous system involvement, when the possible role of microbiota is scrutinized [18,19]. The microbiota composition and, particularly, alterations in the Firmicutes/Bacteroidetes ratio (the two most important bacterial phyla in the gastrointestinal tract) may have repercussions on intestinal barrier permeability, even leading to a condition of “leaky gut”, thereby promoting lipopolysaccharide (LPS) translocation to the brain and release of long-chain fatty acids overproduced by bacterial metabolism. These molecules determine microglia activation and neuroinflammation, in a bidirectional loop between the gut and brain, in the context of a gut–brain axis [20].

In CeD, microbiota composition is skewed towards Gram-negative species such as Escherichia coli and Bacteroides, as opposed to Gram-positive Lactobacilli and Bifidobacteria; overall, both pathogenic and commensal bacteria seem to play a role in gluten metabolism, with a protective role of the latter, particularly Lactobacilli [19]. Bifidobacterium may have a dual role, as it seems to modulate both a Th1 pro-inflammatory milieu in vitro and the production of the anti-inflammatory cytokine IL-10 [19]. On the other hand, in an experimentally induced rhesus macaque model of CeD, several tight junctions and their associated proteins, such as zonulin and haptoglobin-2, known to also be expressed in the human blood–brain barrier, were shown to be dysregulated, along with the peroxisome proliferator activated receptor gamma (*PPARγ*) gene, leading to intestinal inflammation, increased permeability, and dysbiosis [21].

**Figure 2:**
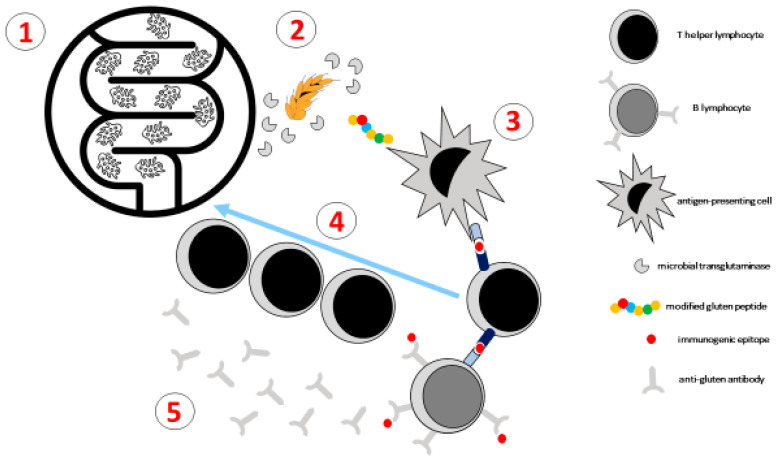
Dysbiosis (1) may lead to bacterial production of microbial transglutaminase (2), whose enzymatic activity may yield immunogenic peptides from gluten, with resulting presentation of gluten epitopes to T cells by antigen-presenting cells (3). T cells become then activated, proliferate, and migrate to the gut (4), where they exert local inflammatory effects; at the same time, T cell–B cell interactions result in the production of anti-gluten antibodies (5).

Moreover, a novel role for microbial transglutaminase (mTG), as a potential environmental inducer of CeD, has recently been proposed. As a member of the transglutaminase family, mTG is functionally similar to tTG, despite a lower molecular weight and a less specific, calcium- and nucleotide-independent activity. Dysbiosis represents a potential source of intraluminal mTg, being secreted by many microbial strains. By crosslinking gluten and gliadin, mTG activity yields immunogenic complexes and neo-epitopes or modified gluten peptides, with possible consequent loss of gluten immune tolerance and onset of autoimmunity [22] (Figure 2). Furthermore, due to its peptide linker and emulsifying activity, it is also used in industrial food processing, including wheat itself, as a non-labeled extraintestinal source.

## 4. Immunological Features: The TCR–Gluten Peptide–MHC Axis

**Figure 3:**
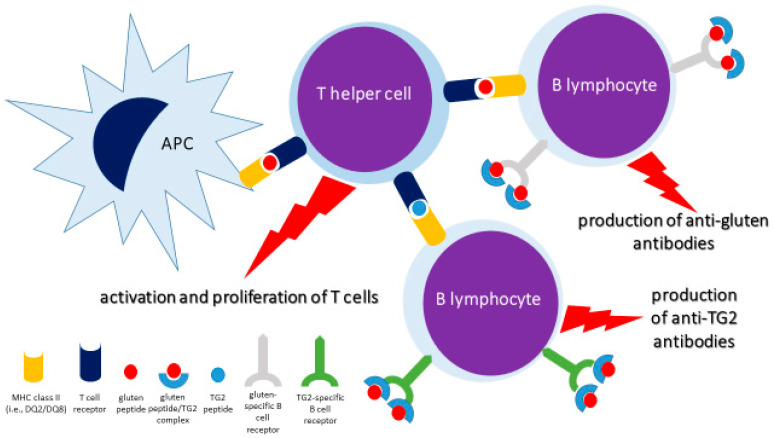
The TCR–gluten peptide–MHC axis. Antigen-presenting cell (APC)-mediated presentation of gluten peptides through MHC class II molecules (i.e., DQ2/DQ8) to helper T cells leads to activation and proliferation of T lymphocytes as well as activation of B lymphocytes specific for gluten or transglutaminase 2 (TG2), with development of plasma cells secreting anti-gluten or anti-TG2 antibodies. Note the hapten-carrier-like role played by the gluten peptide/TG2 complex. For the sake of simplicity, the T helper cell has been depicted with dual T cell receptor specificity, in place of the conventional single selectivity.

The central immunological axis in CeD is the T cell receptor (TCR)–gluten peptide–major histocompatibility complex (MHC) interaction [2]. Gliadins and glutenins in wheat, secalins in rye, and hordeins in barley, all belonging to the class of prolamins and characterized by resistance to digestion by peptidases and proteases, are the main gluten-related proteins responsible for CeD. Following partial breakdown in the upper gastrointestinal tract, the derived oligomers can then interact with APCs expressing MHC class II molecules. In particular, TG2–gluten peptide complexes can be recognized as the pivotal autoantigens that act with a hapten-carrier-like mechanism. Specifically, gluten peptides and TG2–gluten complexes undergo endocytosis by APCs via LDL receptor-related protein 1 (LRP-1) and are transported into the endolysosomal compartment, whereby deamidated gluten peptides are loaded onto disease-associated MHC class II molecules, namely human leukocyte antigen (HLA)-DQ [23,24]. APCs able to present these peptides to pathogenic T cells include dendritic cells (DCs), gluten-specific B cells, and TG2-specific B cells. Then, recognition of MHC class II–peptide complexes by TCRs on disease-specific T CD4+ cells initiates the inflammatory process, with progressive expansion of gluten-reactive T cells, cytokine release, and production of anti-gluten and anti-TG2 antibodies; indeed, concurrently, T cell–B cell interactions lead to the differentiation of plasma cells as well (Figure 3).

Finally, after lymphocyte migration to mesenteric lymph nodes, immune cells gain access to the systemic circulation, thus expanding the organ-specific phase of immune inflammation into a potential multiorgan involvement, possibly explaining the pathogenesis of extraintestinal manifestations of CeD [16,25].

The enterocytes of the duodenum, the major component of differentiated epithelial cells, show increased turnover and proliferation index in inflammatory conditions. They are targeted for immune-mediated killing in CeD, probably owing to the upregulation of both MHC class I chain-related proteins A and B (MICA/B) and HLA-E. MICA/B are induced upon stress, damage, or transformation of cells and act as a “kill me” signal by natural killer (NK) cells; HLA-E is critical for cell recognition by NK cells. Thus, the killed enterocytes become themselves a source of luminal TG2 when extruded [26].

Among gut epithelial cells are the intraepithelial lymphocytes (IELs) [27], which are predominantly composed of TCRαβ+ CD8+ T cells with an effector memory phenotype in non-inflammatory conditions; conversely, during active CeD, TCRγδ+ T cells increase notably in the context of IELs, even with expression of NK cell receptors [28], such as activating NKG2D and CD94/NKG2C [29].

The killing of enterocytes or the action of cytokines may be assumed to be the primary event in CeD, according to the pathogenetic model considered. However, a damaged epithelium and/or genetic alterations in the intestinal barrier or tight junctions, the effects of TG2–gluten complexes on barrier integrity, and/or antigen transcytosis across the gut epithelium by microfold cells (M cells) all may play a role in facilitating the translocation of gluten peptides into the lamina propria. Here, B cells seem to be the most prevalent APCs in active CeD, leading to a characteristic plasmacytosis, with predominance of IgA-expressing plasma cells [30,31].

## 5. The Role of Cytokines in CeD

In this scenario, cytokines may initiate, promote, and amplify the pathogenic events leading to inflammation and tissue damage, in concert with all involved cell populations. In cohorts of susceptible infants, increased serum levels of interferon (IFN)-γ, IL-1β, IL-2, IL-4, IL-6, IL-10, IL-12p70, IL-17A, and TNF-α and a lower Foxp3 and TGF-β expression in peripheral blood mononuclear cells were observed before anti-TG antibody production and at CeD onset. Interestingly, the same study highlighted the non-predictive and protective role of early production of isolated AGAs with respect to anti-DGP and anti-TG [32]. Furthermore, in a mouse model, overexpression of IL-15 both in the epithelium and the lamina propria was demonstrated to be required for the development of villous atrophy in a gluten- and HLA-DQ8-dependent manner and with the fundamental contribution of IFN-γ and TG2 [4]. Other than APCs, epithelial cells have an active role in producing IL-15 in active CeD [33]. IL-15 is able to stimulate an increase in NKG2D receptors on IELs and to induce the NKG2D ligand MICA on epithelial cells themselves, with consequent NK- and T-cell-mediated cytotoxicity; moreover, IL-15 leads to an enhanced production of granzyme B and IFN-γ by IELs, thus boosting lymphocyte killing ability [29]. Additionally, IL-15 shares the common γ-chain receptor subunit and the same target cells as IL-21, which is known to increase the production of IFN-γ and perforins by IELs but also the secretion of metalloproteinases by stromal cells; IL-21 production also appears to correlate with anti-TG2 antibodies and tissue damage [34]. Conversely, IL-2 appears to be significantly augmented after gluten oral challenge and seems to correlate symptomatically with nausea and vomiting [35], thus representing an interesting link between molecular and clinical features. Finally, studies on global gene expression and gene network cluster analysis have shown involvement of Th1, Th2, and Th17 differentiation and signaling pathways in CeD, albeit further clarification is needed [36].

## 6. Diagnostic Issues

Overall, the immunological events result in the well-known histologic alteration of CeD, from infiltrative and hyperplastic to destructive lesions, corresponding to type 1, type 2, and type 3 Marsh classifications, respectively [37,38,39,40].

From a diagnostic point of view, when anti-TG2 IgA positivity is confirmed by the presence of anti-endomysial antibodies, biopsy of the duodenum still remains the gold standard for CeD diagnosis, at least in adults. However, apart from the possibility of rare seronegative forms, a correct diagnosis may be hindered by the extensive differential diagnosis of the many conditions resembling CeD in adults. Specifically, among infectious diseases, the following may simulate CeD: parasitic infestation and giardiasis, Helicobacter pylori infection, tropical sprue, Whipple’s disease, bacterial overgrowth, tuberculosis, HIV, and viral gastroenteritis; among drug-induced forms: olmesartan and angiotension receptor blockers, cytotoxic drugs, immune modulatory drugs, and immune checkpoint inhibitors; among immune/autoimmune and inflammatory conditions: common variable immunodeficiency, sarcoidosis, eosinophilic gastroenteritis, inflammatory bowel disease, collagenous enteritis, lymphoma, food protein enteropathies, and gluten sensitivity [41].

When considering clinical manifestations, diagnosis may not be straightforward because of atypical presentations.

Gastrointestinal symptoms are related to mucosal damage and the consequent malabsorptive syndrome. In classic CeD, postprandial abdominal pain, chronic diarrhea, bloating, steatorrhea, weight loss, weakness, nausea, and vomiting may be the predominant features; these symptoms may be missing at the onset or preceded by extraintestinal manifestations in atypical presentations [7]. In children, failure to thrive may be indicative of CeD; oligoarticular and asymmetric arthritis may sometimes be the presenting manifestation. Atypical symptoms may also include epistaxis, vertigo, nystagmus, obstructive sleep apnea, nasal septal perforation, and sensorineural hearing loss, the latter probably due to both malnutrition and neuroimmune-mediated mechanisms [42,43].

CeD is being diagnosed more frequently but is often undiagnosed when encountered by surgeons. Indeed, CeD patients are at an increased risk for surgery for common problems such as appendicitis. Patients with undiagnosed CeD undergoing surgery may develop symptoms leading to diagnosis postoperatively; for instance, undiagnosed CeD should be suspected if malabsorptive symptoms develop following surgery [44].

A possible correlation with recurrent miscarriages may result from intestinal malabsorption, a state of systemic inflammation, or CeD antibodies interfering with endometrial angiogenesis [45]. Often, anemia and osteoporosis can precede the diagnosis of CeD [46]. Anemia is the most frequent extraintestinal manifestation, being the combination of iron deficiency due to malabsorption and anemia of chronic diseases. Most patients recover from anemia during a gluten-free diet, however, poly/hypermenorrhea may result in persistent hypochromic and microcytic anemia [47]. Other types or causes of anemia are less common [48,49,50].

Osteoporosis is primarily related to malabsorption of calcium and vitamin D [51]. Consequently, hypocalcemia-induced hyperparathyroidism may lead to osteoclast hyperactivity, with reduction of bone mineral density, microarchitecture disruption, and an increased risk of fracture in CeD [52]. The pro-inflammatory cytokines IL-1, IL-6, and TNF-α, which are increased in the serum of CeD patients, are known to be involved in the enhanced expression of receptor activator of nuclear factor kappa-B ligand (RANKL), its binding with RANK on osteoclast precursors, and the inhibition of osteoprotegerin (OPG). All these events lead to bone resorption [53]. Although a gluten-free diet can revert the events leading to bone resorption, a subgroup of patients may not normalize bone mineral density, thus still showing bone loss on long-term gluten abstinence [54].

## 7. Genetic Issues

Currently, the average prevalence of CeD is estimated to be around 1% [55]. HLA-DQ2 and DQ8 are necessary but not sufficient for disease onset [56]. Indeed, the prevalence of HLA-DQ2/HLA-DQ8 haplotypes is around 35–40% in the general population, but only 3% of these susceptible individuals develop CeD [57]. Moreover, as only 55% of CeD cases are traced down to heritability, genetic polymorphisms and genomic variations, including non-HLA-related genes, may play a role, as suggested by genome-wide association studies (GWASs) [58]. For these reasons, screening for HLA genetic susceptibility is not recommended in the diagnostic algorithm of CeD. Conversely, other environmental non-gluten factors have been scrutinized as possible contributors to disease development, particularly viral agents, such as rotavirus, adenovirus, enterovirus, and Epstein–Barr virus, as well as Helicobacter pylori [59,60,61].

Enteropathy-associated T-cell lymphoma (EATL) may complicate CeD, particularly in cases of persisting villous atrophy. Rarely, the persistence or recurrence of symptoms, malabsorption, and villous atrophy, despite a strict gluten-free diet, may be the expression of refractory celiac disease, particularly in patients over 50 years old. The presence of at least 20% of IELs with an aberrant immunophenotype distinguishes type 2 from type 1 refractory CeD. In type 1, the characteristic exogenous gluten dependence of the disease is lost to become a “fully” dysimmune gluten-independent condition; type 2, on the other hand, seems to be more frequently associated with homozygosity of HLA-DQ2 and can be considered as an in situ T cell lymphoma at high risk of EATL progression [62,63].

## 8. Malignancies and CeD

In spite of controversial findings, the association between CeD and malignancies is reported in many studies, particularly in males older than 40 years and during the first years of diagnosis. In a Swedish population-based cohort study of 49.829 patients, indeed, a slightly but significantly increased risk of mortality from all causes and from specific cardiovascular, neoplastic, or respiratory causes was found, mostly in the first year of CeD diagnosis [64]. Correlations were found with gastroesophageal cancer, hepatobiliary and pancreatic cancer, thyroid papillary cancer, and small bowel cancer, while risk was low for colorectal cancer. CeD shares with these malignancies such hallmarks as inflammation, genome instability and mutations, leukocyte telomere shortening, epigenomic modifications, epigenetic reprogramming, and alteration of gut microbiota, with consequent epithelial transdifferentiation and dedifferentiation, immune evasion, and tumor growth and invasiveness [65,66]. On the contrary, female gender-related cancers such as breast, endometrial, and ovarian neoplasms seem to be less frequent, probably due to CeD-associated low estrogen exposure, early menopause, lower body mass index, or concomitant lactose intolerance, with reduced intake of insulin-like growth factor-1 from milk products [67]. With regard to male-related cancers, an increased risk of prostate as well as lung cancer during CeD has not been found [68,69,70], while relevant data of association with testis cancer are not available.

## 9. Autoimmune Diseases and CeD

Notably, the main genetic (MHC) predisposition factors for CeD are located on chromosome 6p21, which also harbors immune-related genes, whose dysregulation is known to predispose to autoimmune diseases [55]. The genetic correlations between CeD and many and/or multiple autoimmune diseases have been highlighted by a recent study through Mendelian randomization [71].

A link with certain autoimmune diseases, such as primary biliary cholangitis, autoimmune hepatitis, primary sclerosing cholangitis, and autoimmune thyroid diseases, has been recognized in the increased intestinal permeability, the amplification of inflammation through portal circulation, the molecular mimicry between bacterial antigens and epitopes recognized by antimitochondrial antibodies, the aberrant T cell homing to the liver, and tTG reactivity with thyroid tissue in CeD patients, depending on the specific autoimmune condition considered [72].

Patients affected by Hashimoto’s thyroiditis and Graves’ disease show a higher prevalence of CeD, while the association between CeD and type 1 diabetes is supported by a significant correlation between anti-GAD antibodies, anti-thyroglobulin antibodies, and CeD [73]. Among rheumatic diseases, rheumatoid arthritis and systemic lupus erythematosus have been associated with CeD [74].

CeD, in combination with autoimmune thyroid diseases or other autoimmune conditions, can be classified into type 3 or type 4 multiple autoimmune syndrome, respectively [75].

Finally, the prevalence of IgA deficiency among patients with CeD is at least 5–15 times higher than that of the general population [76].

## 10. Nutritional Considerations

Gluten commonly refers to the prolamins (the main storage proteins) of wheat, rye, and barley, which are pathogenic in patients with CeD. Wheat gluten is composed of alcohol-soluble gliadins (namely, α-gliadins, γ-gliadins, and ω-gliadins) and alcohol-insoluble glutenins (categorized as high-molecular-weight and low-molecular-weight glutenins). Gliadins and glutenins are characteristically enriched in proline and glutamine amino acids; because of the high proline content, these proteins show resistance to proteolytic activity by enzymes in the gastrointestinal tract. Hence, the long gliadin peptides generated in the gastrointestinal tract become able to trigger harmful immune responses in patients with CeD [1,2,3,4].

Consequently, the cornerstone of CeD treatment is a gluten-free diet, which involves no wheat, rye, or barley proteins. Fortunately, gluten is a protein with limited nutritional value, which can be substituted for by other dietary proteins. Since wheat is the main ingredient of bread, pasta, pastries, and snack foods, its complete avoidance can be very difficult. Although strict gluten avoidance is crucial for treatment of patients with CeD, the nutritional composition of gluten-free diets may be unbalanced, leading to a poor intake of iron and fibers [77]. In addition, they may result in reduced consumption of whole grains beneficial for cardiovascular health, with a consequent increase in cardiovascular disease risk [78]. On the other hand, acceptable grains include rice, oats, buckwheat, corn, millet, and quinoa [15].

Although oats contain < 20 parts per million gluten (the standard definition for a gluten-free diet [79]), their inclusion in gluten-free diets has been a controversial issue. Oats are generally well tolerated by most patients with CeD, showing no harmful effects on small intestinal mucosal morphology or clinical symptoms, even after long-term consumption. Thus, oats uncontaminated by gluten are safe for almost all patients with CeD. However, some patients have been reported to experience clinical symptoms and mucosal inflammation following consumption of oats, possibly because of gluten contamination. Notwithstanding, adding oats into gluten-free diets may yield several nutritional benefits, being a source of soluble fibers, minerals, and vitamins, and because of their ability in lowering postprandial blood glucose and low-density lipoprotein levels [80].

Currently, a wide selection of gluten-free wheat substitutes with good palatability is available for CeD patients, making gluten-free diets more acceptable by patients. Several tasty and nutritious flours can be made as substitutes for wheat flour or whole wheat using amaranth, barley, coconut, chestnut, maize, millet, teff, oat, rye, sorghum, soy, and rice. In addition, amaranth and quinoa can be good vitamin sources, sorghum is enriched in thiamine, and millet also provides carotenoids. Thus, these wheat substitutes can improve the nutritional quality of gluten-free products, by providing proteins, healthy fats, fibers, and minerals [81]. Moreover, these flours have also been found to improve the physicochemical, pasting, and rheological properties of the doughs [82].

In addition, modern industrial preparations of gluten-free foods are enriched with minerals and vitamins, in order to avoid nutritional deficiencies. Previously, in fact, gluten-free flours were not supplemented with nutrients (e.g., iron and B vitamins), thus leading to nutritional deficiencies in patients adhering to a strict gluten-free diet. The most frequently described deficiencies in CeD patients involve iron, vitamin D, calcium, vitamin B12, folate, and zinc. It is also important to comply with a lactose-deficient diet (i.e., milk, fresh cheese, cream, ice cream) during the first weeks of a gluten-free diet.

In fact, lactose intolerance is common in CeD patients, owing to the depletion of intestinal lactase secondary to gut inflammation [6,7]. Such a restriction may be lifted once the intestinal lactase levels are restored, following mucosal healing (unless the patient is lactose-intolerant as well, in which case fresh dairy products should be avoided indefinitely). However, CeD patients may continue consuming yogurt, aged cheese, and kefir, which are known to be naturally low in lactose. For the same reasons, patients should be checked for blood vitamin D levels and bone density. Gut inflammation may cause low serum levels of vitamin D, prompting addition of calcium and vitamin D to the diet [6,7]. Nevertheless, dietary sources may provide sufficient bioavailable calcium, therefore their consumption should be part of a balanced diet. Apart from vitamin D, the other fat-soluble vitamins (A, E, K) may also be poorly absorbed; the same is true for B vitamins and minerals, including magnesium, zinc, and selenium. Thus, vitamin and mineral replacement is required as well until mucosal recovery. Finally, red meat, poultry, and fish represent a source of bioavailable iron and their consumption should also be encouraged.

However, adherence to a gluten-free diet is dependent on a high level of motivation in patients; therefore, a diet completely devoid of gluten is very difficult to maintain. Notwithstanding, a daily intake of 10–20 mg of gluten seems to be harmless, whereas daily consumption of >200–500 mg may induce a small amount of intestinal villous damage and inflammation [83,84]. For the above reasons, consultation with a nutritionist is warranted for optimal management of CeD patients. As already anticipated, the nutritional composition of gluten-free products may be unsatisfactory [77,78], as they may be unbalanced and not fortified with micronutrients. Ideally, nutritionists with a special interest and experience in CeD would be highly desirable in order to thoroughly assess dietary nutrient deficiencies and to educate patients on how to maintain a strict gluten-free diet, providing at the same time counseling for healthy alternatives to gluten. A gluten-free diet should be balanced with proper iodine, iron, calcium, and vitamin D, among others, upon nutritionist advice.

Finally, novel therapeutic approaches, if successful, may help ease management of CeD patients. Oral enzyme supplements aimed at eliminating hidden gluten contamination, enhancement of mucosal tight junction integrity, TG2 inhibition, and gluten immunotherapy are some of the experimental therapies being evaluated [85].

## Data Availability

Not applicable.
